# Identification of a novel metabolic and lipidomic signature for 90-day functional outcome after ischemic stroke: an exploratory proof-of-concept study

**DOI:** 10.3389/fneur.2026.1931495

**Published:** 2026-09-03

**Authors:** Tingwei Wang, Jian Ji, MaiQiu Wang, Rongli Fan, Yongli Ye, Shuang Zhang, Shengfang Wu, Lei Zhang, Jiajia Fang

**Affiliations:** 1 College of Biological & Chemical Engineering, Zhejiang University of Science and Technology, Hangzhou, Zhejiang, China; 2 Zhejiang Key Laboratory of Biomedical Intelligent Computing Technology, Hangzhou, Zhejiang, China; 3 School of Food Science and Technology, International Joint Laboratory on Food Safety, Synergetic Innovation Center of Food Safety and Quality Control, Jiangnan University, Wuxi, Jiangsu, China; 4 Jiangnan University Instrumental Analysis and Laboratory Animal Center, Wuxi, Jiangsu, China; 5 Department of Neurology, The Fourth Affiliated Hospital, Zhejiang University School of Medicine, Yiwu, Zhejiang, China

**Keywords:** biomarkers, ischemic stroke, lipidomics, metabolomics, prognosis

## Abstract

**Background:**

Ischemic stroke is a leading cause of death and disability worldwide, yet the systemic metabolic mechanisms underlying poor functional recovery remain incompletely understood. Dysregulated lipid metabolism is a key pathogenic factor, and advances in metabolomics and lipidomics provide new opportunities for discovering pathophysiological signatures.

**Methods:**

In this exploratory study, we integrated serum metabolomic and lipidomic profiling in 44 patients with acute ischemic stroke, stratified by favorable (*n* = 23) or unfavorable (*n* = 21) 90-day functional outcomes. Differential metabolites and lipids were identified, pathway-related molecular alterations were characterized, and candidate markers were evaluated to establish a proof-of-concept metabolic signature.

**Results:**

Patients with unfavorable outcomes exhibited global metabolic reprogramming characterized by structural lipid disassembly (widespread reductions in membrane phospholipids, including phosphatidylcholines and ether phospholipids), mitochondrial energy failure (acylcarnitine abnormalities consistent with impaired *β*-oxidation), and antioxidant depletion (marked by decreased cis-caffeic acid). An exploratory three-marker panel—the C8:2-OH(3)/C18:2-OH(3) ratio, the free DHA/PC(44:12) ratio, and cis-caffeic acid—showed promising discriminatory potential in this derivation cohort (AUC = 0.950). Adding this metabolic panel to the initial NIHSS score improved the AUC to 0.975, suggesting incremental value beyond clinical assessment alone.

**Conclusion:**

Integrated serum metabolomics and lipidomics identified a metabolic signature reflecting lipotoxicity, mitochondrial dysfunction, and oxidative stress. These preliminary findings highlight systemic metabolic reprogramming as a potential driver of unfavorable outcome and provide a proof-of-concept for combining molecular phenotyping with conventional clinical scales. Without external validation and multiple-testing correction, these results should be viewed as hypothesis-generating.

## 1 Introduction

Stroke is characterized by acute neurological deficits caused by focal brain injury and is one of the leading causes of death and disability worldwide (1). According to the Global Burden of Disease study, approximately 11.95 million new stroke cases occur each year, and stroke-related mortality and disability continue to increase (2). In China, the latest national guidelines for the prevention and treatment of cerebrovascular diseases report approximately 3.94 million new stroke cases annually, accounting for nearly one-third of the global total. Ischemic stroke is the predominant subtype, representing approximately 72% of all cases (3). More than 28 million stroke survivors currently live in China, imposing a substantial social and economic burden. Ischemic stroke results from cerebral vascular occlusion and has diverse etiologies, including atherosclerosis, cardioembolism, and small-vessel disease (4). In recent years, the incidence of ischemic stroke has steadily increased among young and middle-aged adults, particularly those aged 35–55 years. This trend is driven by the increasing prevalence of early-onset hypertension, diabetes mellitus, obesity, and unfavorable lifestyle factors (5).

Despite major advances in the acute management of ischemic stroke, approximately 75% of survivors continue to suffer from residual neurological deficits within the first year. These deficits affect sensory, language, and cognitive domains and may be accompanied by multiple secondary complications (6). Even after long-term follow-up of 3–4 years, only 62% of patients achieve complete functional independence (7). Among these sequelae, long-term neurological and functional impairments are among the most common and disabling complications affecting recovery and quality of life (8, 9). Current prognostic assessment after ischemic stroke relies primarily on neurological scales, such as the National Institutes of Health Stroke Scale (NIHSS) and the modified Rankin Scale (mRS), as well as neuroimaging examinations (10). However, the systemic molecular mechanisms driving these divergent recovery trajectories remain poorly defined, and blood-based biological signatures that can capture this systemic exhaustion at an early stage remain limited (11).

Lipids are essential structural components and functional regulators of the central nervous system (CNS), accounting for approximately 50–60% of brain dry weight (12). Multiple lipid classes, including phosphatidylcholines (PCs), triglycerides (TGs), ceramides, and cholesterol, are involved in the onset, progression, and recovery of ischemic stroke. Brain injury and systemic stress responses can further disrupt systemic lipid homeostasis (13). Therefore, abnormal levels of specific serum lipids, including distinct phospholipid species, may provide a non-invasive window for monitoring brain health (14). Previous studies have shown that impaired high-density lipoprotein cholesterol efflux capacity is associated with poor stroke outcomes. Patients who die or experience unfavorable functional outcomes within 1 year also show significantly elevated levels of oxidized low-density lipoprotein (15, 16). However, the precise associations between specific lipid molecules and ischemic stroke prognosis, as well as the underlying mechanisms, remain unclear.

Recent advances in untargeted metabolomics and lipidomics have provided new opportunities to characterize systemic metabolic dysregulation and identify pathophysiological signatures (13). The pathogenesis of functional outcome after ischemic stroke involves complex reprogramming of metabolic and lipid networks that cannot be fully captured by single-omics analyses. In this exploratory study, we integrated serum metabolomics and lipidomics to systematically characterize the perturbations associated with unfavorable outcomes after ischemic stroke. Rather than building a traditional clinical prediction model, we aimed to derive a metabolic signature from key molecular ratios that could provide insights into systemic pathological drivers such as lipid disassembly and energy failure, and to evaluate whether these molecular alterations offer incremental biological information beyond conventional clinical scales like the NIHSS.

## 2 Materials and methods

### 2.1 Patient population

This prospective cohort study included 44 patients with acute ischemic stroke who were hospitalized at the Fourth Affiliated Hospital of Zhejiang University, Zhejiang Province, China, between January 2020 and December 2023. The inclusion criteria were admission within 72 h of symptom onset, diagnosis of acute ischemic stroke, and age ≥18 years. The exclusion criteria were a pre-existing psychiatric disorder, such as schizophrenia or depression; a history of other neurological disorders; aphasia preventing completion of scale assessments; missing mRS follow-up data; or severe comorbidities, such as myocardial infarction or end-stage renal failure.

The Ethics Committee of the Fourth Affiliated Hospital of Zhejiang University approved this study. The protocol complied with the “Measures for Ethical Review of Biomedical Research Involving Human Beings,” the “International Ethical Guidelines for Health-related Research Involving Humans,” and the Declaration of Helsinki. The study also followed international ethical standards, including Good Clinical Practice (GCP) and the International Council for Harmonisation (ICH) Guideline for Good Clinical Practice, as well as applicable domestic laws and regulations. Patients were screened strictly according to predefined inclusion and exclusion criteria.

Initially, 146 patients with cerebrovascular disease were screened. We sequentially excluded patients with hemorrhagic stroke or post-infarction hemorrhage (*n* = 28), Moyamoya disease (*n* = 1), severe primary kidney/liver diseases or leukemia (*n* = 21), those admitted outside the 72-h window or lacking blood samples (*n* = 34), and those lost to follow-up or missing complete 90-day mRS assessment (*n* = 18). The final cohort consisted of 44 patients with acute ischemic stroke. The detailed patient screening and inclusion process is illustrated in Supplementary Figure S1.

### 2.2 Blood sample collection, processing, and laboratory testing

Peripheral venous blood samples were collected from all patients at emergency admission and divided into two aliquots. For metabolomic and lipidomic analyses, one aliquot was immediately centrifuged, transferred into sterile tubes, and stored at −80 °C until further processing. The remaining aliquot was sent immediately to the hospital laboratory for routine testing, including complete blood count (CBC), liver and renal function tests, electrolyte measurement, coagulation parameters, and other relevant biomarkers.

### 2.3 Functional outcome assessment

Functional outcome at 90 days after symptom onset was assessed using the mRS. Patients with an mRS score of 0–2 at 90 days were assigned to the favorable outcome group, whereas those with an mRS score >2 were assigned to the unfavorable outcome group.

### 2.4 Metabolite extraction and analysis

Metabolites were extracted and analyzed according to the standard operating procedures (SOPs) established by the Fiehn laboratory. Briefly, 40 μL of serum was extracted with 1 mL of extraction solvent [acetonitrile/isopropanol/water (3:3:2, v/v/v)]. The mixture was vortex-mixed and centrifuged. A 450 μL aliquot of the metabolite extract was dried for 6 h using a Savant High-Capacity SpeedVac Plus concentrator (Thermo Fisher Scientific, USA). The dried extract was then treated with 80 μL of 20 mg·mL^−1^ methoxyamine hydrochloride and incubated at 80 °C in a metal bath for 30 min. After cooling to room temperature, 100 μL of N-methyl-N-(trimethylsilyl)trifluoroacetamide (MSTFA; containing 1% trimethylchlorosilane, v/v) was added to the reaction system, followed by incubation at 70 °C for 40 min.

After derivatization, a fatty acid methyl ester (FAME) mixture (C8–C16: 1 mg/mL; C18–C24: 0.5 mg/mL in chloroform) was added as the internal standard for retention time locking and quantification. The derivatized samples were analyzed within 24 h.

Metabolites were detected by GC-TOF/MS (LECO, USA). Samples were injected at 250 °C with an injection volume of 1 μL and a split ratio of 10:1. The carrier gas was operated in constant-flow mode at 1 mL min^−1^. Chromatographic separation was performed on a DB-5MS column (30 m × 0.25 mm inner diameter, 0.25 μm film thickness; Agilent). The oven program was as follows: initial temperature, 80 °C; initial hold, 4 min; ramp rate, 6 °C min^−1^; final temperature, 300 °C; and final hold, 10 min. The MS conditions were as follows: ionization mode, electron impact (EI); emission current, 1 mA; electron energy, −70 eV; interface temperature, 280 °C; and ion source temperature, 230 °C. MS data were acquired in full-scan mode over an m/z range of 50–650 at a scan rate of 20 spectra/s. Raw data were converted to ABF format using ABF Converter (https://www.reifycs.com/AbfConverter/index.html). MS-DIAL software, together with the Fiehn library, was used for peak extraction, noise filtering, baseline correction, peak alignment, deconvolution, peak annotation, and peak-height integration. For metabolomic quantification, the peak heights of target metabolites were normalized to the corresponding internal standards (FAMEs) and expressed as relative abundances.

### 2.5 Lipidomic analysis

Serum lipids were extracted according to the SOPs established by the Fiehn laboratory. Briefly, 40 μL of serum was extracted with 1 mL of extraction solvent [methyl tert-butyl ether/methanol/water (65:22:16, v/v/v)]. The mixture was vortex-mixed and centrifuged at 12,000 × g for 10 min. A 450 μL aliquot of the supernatant was transferred to a new centrifuge tube and dried using a Savant High-Capacity SpeedVac Plus concentrator. The dried lipid extract was reconstituted in 200 μL of reconstitution solvent [acetonitrile/isopropanol/water (65:30:5, *v/v/v*)]. Before lipid extraction, an internal standard mixture was added to each sample. The mixture contained lysophosphatidylcholine (LPC) (19:0, 0.28 μg/mL), sphingomyelin (SM) (12:0, 0.28 μg/mL), deoxycholic acid-d4 (0.56 μg/mL), triacylglycerol (TAG) (45:0, 0.94 μg/mL), FA C16:0-d3 (0.94 μg/mL), and FA C18:0-d3 (0.94 μg/mL).

Serum lipidomic analysis was performed using a pseudo-targeted approach, as described by Xu et al. (17). A Q-Trap 5,500 mass spectrometry system (Sciex, USA) was used for pseudo-targeted lipidomic profiling. Chromatographic separation was performed on a BEH C8 column (100 × 2.1 mm, 1.7 μm particle size; Phenomenex) maintained at 55 °C, with an injection volume of 2 μL. The liquid chromatography gradient elution program and mass spectrometry parameters are provided in Supplementary Table S1. Data were processed using SCIEX OS Software 2.0 to quantify lipids monitored by the pseudo-targeted lipidomic method.

For lipidomic quantification, the peak heights of individual lipid species were normalized to their corresponding class-specific internal standards to yield relative abundances. Specifically, PC(44:12) and other phosphatidylcholines were quantified relative to the internal standard LPC(19:0). In the lipid nomenclature used here, “O-” denotes an alkyl-ether linkage (e.g., PC(O), PE(O)), whereas “P-” denotes a vinyl-ether linkage characteristic of plasmalogens (e.g., PE(P)).

### 2.6 Statistical analysis

Statistical analyses were performed using R software and SPSS version 20.0. Continuous variables were evaluated for normality using the Shapiro–Wilk test. Normally distributed variables were presented as mean ± standard deviation (SD) and compared using Student’s t-test. Skewed continuous variables were presented as median [interquartile range, IQR] and compared using the Mann–Whitney U test. Categorical variables were presented as n (%) and compared using the Chi-square test or Fisher’s exact test. To assess independent prognostic value, multivariable binary logistic regression was performed. Given the exploratory nature of this study, nominal *p*-values were used without stringent false discovery rate (FDR) corrections. A two-sided *p* < 0.05 was considered statistically significant. Differences between the area under the receiver operating characteristic curves (AUCs) were statistically compared using DeLong’s test.

## 3 Results

### 3.1 Baseline clinical and biochemical characteristics between the favorable and unfavorable outcome groups

**Figure 1:**
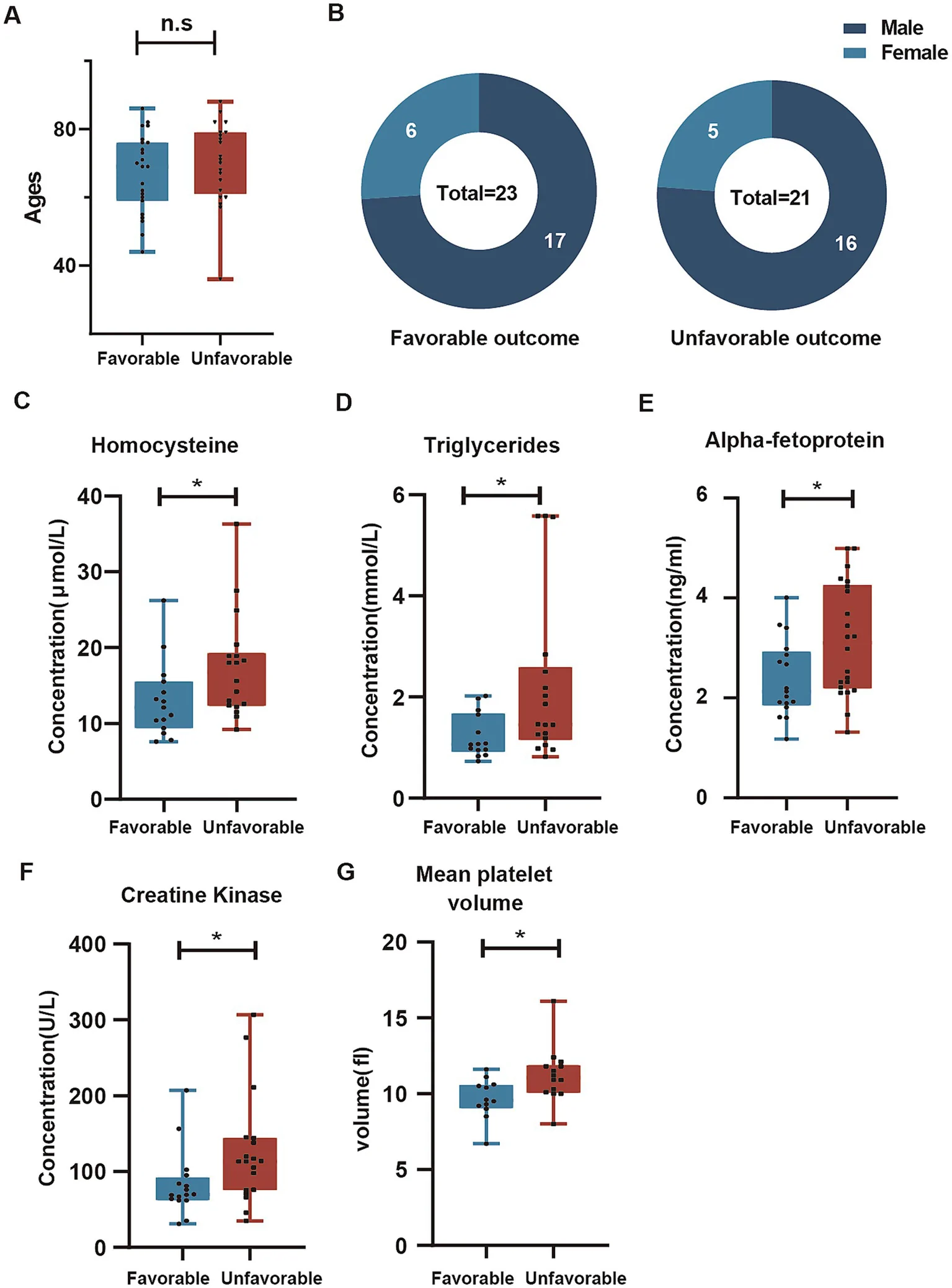
Comparison of clinical characteristics between patients with favorable outcome and unfavorable outcome after stroke. **(A)** Age distribution in the two groups. **(B)** Sex composition (male/female). **(C–H)** Clinical indicators showing significant differences between groups, including homocysteine, triglycerides, alpha-fetoprotein, creatine kinase, and mean platelet volume. Data for normally distributed variables are presented as mean ± SEM. Data for non-normally distributed variables are presented as median with interquartile range. *p < 0.05.

Based on modified Rankin Scale (mRS) scores and clinical manifestations, the 44 enrolled patients were classified into a favorable outcome group (mRS ≤ 2, *n* = 23) and an unfavorable outcome group (mRS > 2, *n* = 21). As detailed in Table 1, baseline demographic and clinical features were well-balanced between the two groups. There were no significant differences in age (67.22 ± 11.48 vs. 70.10 ± 12.09 years, *p* = 0.423) or sex distribution (*p* = 0.937) (Figures 1A,B). Crucially, key clinical confounders—including the prevalence of hypertension and diabetes mellitus, admission glucose levels, long-term glycemic control (HbA1c), and pre-admission use of antihypertensive or antiplatelet agents—showed no statistically significant differences between the groups (all *p* > 0.05). Notably, none of the 44 patients in this cohort received acute reperfusion therapy (intravenous thrombolysis or endovascular thrombectomy) due to delayed presentation, effectively eliminating this major therapeutic confounder.

**Table 1:** Baseline clinical and biochemical characteristics of patients with acute ischemic stroke according to 90-day functional outcome.

Variables	Favourable outcome (mRS 0–2) (*n* = 23)	Unfavourable outcome (mRS > 2) (*n* = 21)	*p*-value
Demographics and clinical features			
Age, years (Mean ± SD)	67.22 ± 11.48	70.10 ± 12.09	0.423
Male sex, *n* (%)	17(73.9)	16(76.2)	0.937
Initial NIHSS score, median [IQR]	1[0, 1.5]	5[3, 8.75]	<0.0001
Reperfusion therapy (IVT/EVT), *n* (%)	0 (0%)	0 (0%)	N/A
Hypertension, *n* (%)	10 (43.5%)	14 (66.7%)	0.129
Diabetes mellitus, *n* (%)	1(4.3%)	1(4.8%)	1.000
Admission glucose, mmol/L (Mean ± SD)	5.57 ± 2.90	5.81 ± 2.63	0.806
HbA1c, % (Mean ± SD)	7.54 ± 1.88	7.61 ± 1.31	0.937
Medications prior to admission			
Antihypertensive agents, *n* (%)	7 (30.4%)	9 (42.9%)	0.404
Antiplatelet agents, *n* (%)	0 (0%)	3 (14.3%)	0.060
Antidiabetic agents, *n* (%)	1 (4.3%)	1 (4.8%)	1.000
Laboratory parameters			
TG, mmol/L, median [IQR]	1.065[0.9525, 1.65]	1.45[1.21, 2.59]	0.027
HCY, μmol/L, median [IQR]	12.1[9.9, 15.15]	15.6[12.45, 18.9]	0.029
CK, U/L, median [IQR]	69.75[63.5, 89.5]	113.7[75.95, 144]	0.018
AFP, ng/mL, median [IQR]	2.13[1.89, 2.76]	3.22[2.24, 4.17]	0.017
MPV, fl, median [IQR]	9.55[9.15, 10.55]	11.2[10.15, 11.8]	0.014
ESR, mm/h, median [IQR]	15.5[8, 23]	10[6, 14.25]	0.194

Continuous variables with normal distribution are presented as mean ± standard deviation (SD) and were compared using Student’s *t*-test. Non-normally distributed variables (including initial NIHSS score and all laboratory parameters) are presented as median [interquartile range, IQR] and were compared using the Mann–Whitney *U*-test. Categorical variables are presented as *n* (%) and were compared using the Chi-square test or Fisher’s exact test, as appropriate. NIHSS, National Institutes of Health Stroke Scale; IVT, intravenous thrombolysis; EVT, endovascular thrombectomy; HbA1c, glycated hemoglobin; TG, triglyceride; HCY, homocysteine; CK, creatine kinase; AFP, alpha-fetoprotein; MPV, mean platelet volume; ESR, erythrocyte sedimentation rate.

As expected, the initial NIHSS score was significantly higher in the unfavorable outcome group compared to the favorable outcome group (*p* < 0.001). Regarding laboratory parameters, compared with the favorable outcome group, patients in the unfavorable outcome group exhibited significantly higher levels of homocysteine (HCY), triglycerides (TG), alpha-fetoprotein (AFP), creatine kinase (CK), and mean platelet volume (MPV) (all *p* < 0.05) (Figures 1C–G).

### 3.2 Comparison of serum metabolomics of ischemic stroke patients

**Figure 2:**
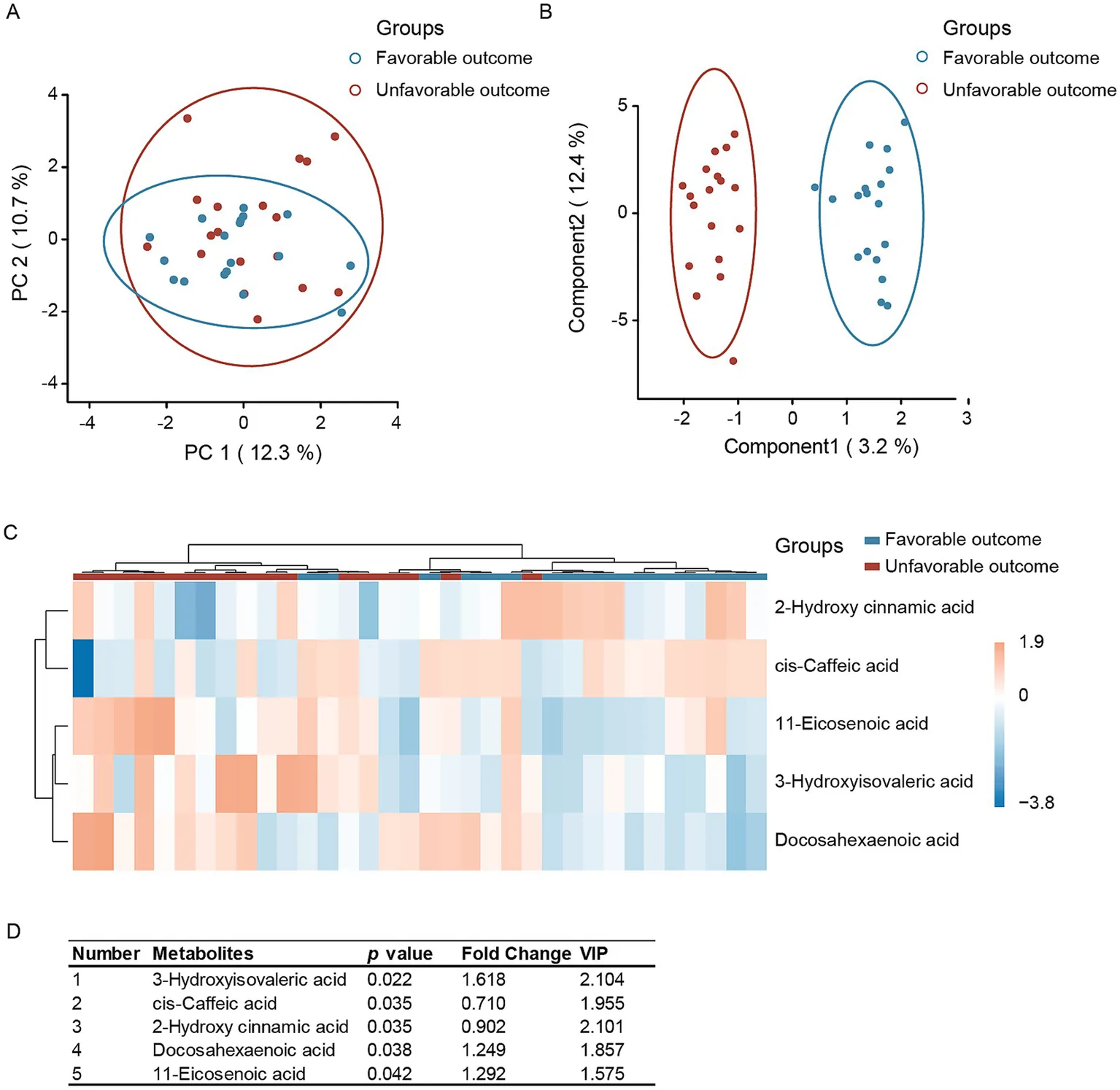
Metabolomic profiling of ischemic stroke patients with favorable versus unfavorable outcome. **(A)** PCA plot illustrating the major sources of metabolite variance. **(B)** Orthogonal partial least squares discriminant analysis (OPLS-DA) score plot comparing the favorable outcome and unfavorable outcome groups. **(C)** Heatmap of differential metabolites generated via hierarchical Pearson clustering (selection criteria: *p* < 0.05, VIP > 1). **(D)** Statistical analysis of differential metabolites.

**Figure 3:**
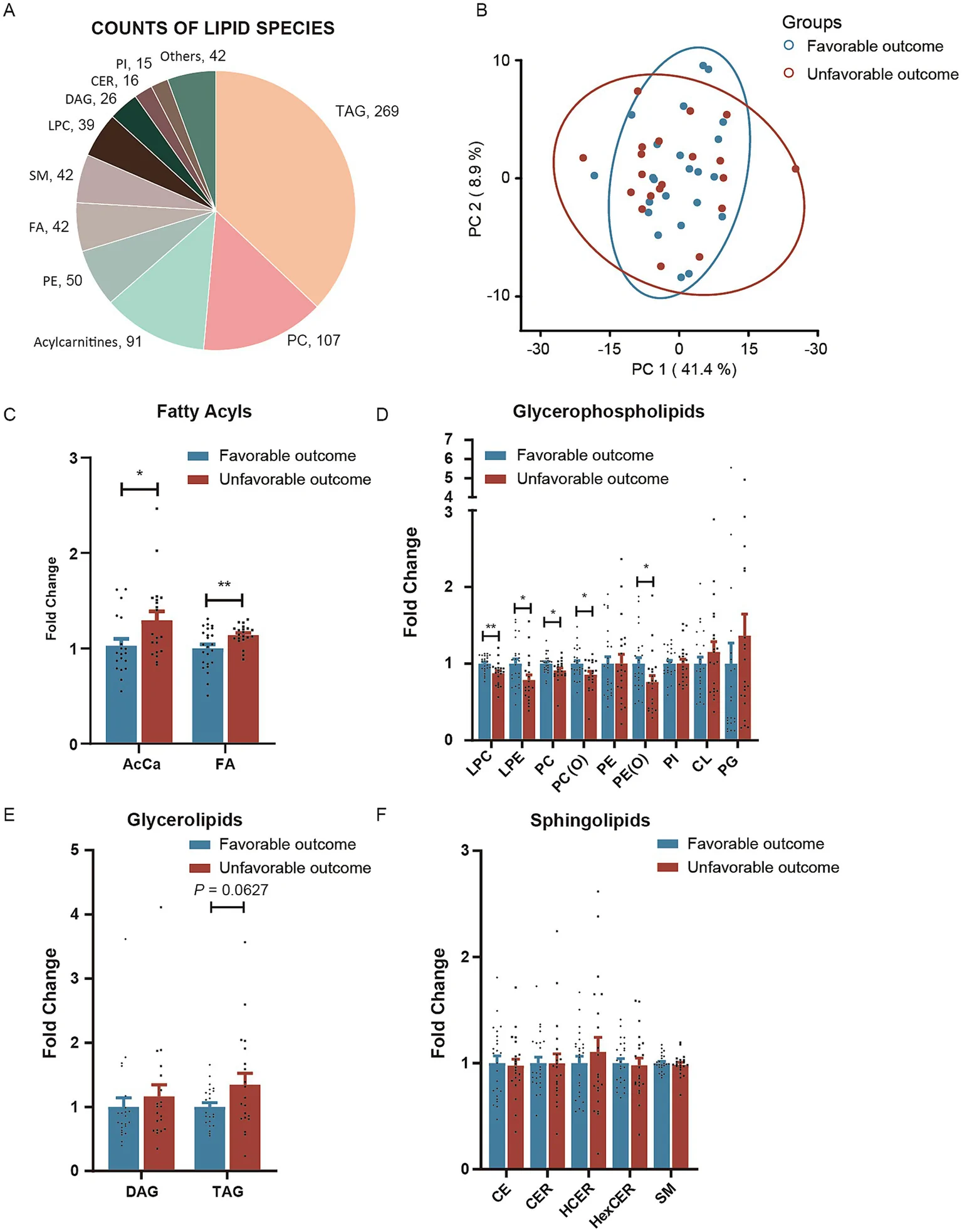
Alterations in global lipid composition and distribution between patients with favorable and unfavorable outcome. **(A)** Distribution of all lipid classes detected. **(B)** PCA plot illustrating the major sources of lipid variance. **(C–F)** Intensity fold change of fatty acyls **(C)** fatty acyls, **(D)** glycerophospholipids, **(E)** glycerolipids, and **(F)** sphingolipids. “O-” denotes an alkyl-ether linkage, and “P-” denotes a vinyl-ether linkage characteristic of plasmalogens.

Given the differences in multiple biochemical indices between the two groups, we performed serum metabolomic profiling to characterize outcome-related metabolic alterations. A total of 73 metabolites were successfully identified and quantified by GC-TOF/MS. Principal component analysis (PCA) was performed, and the score plots for the two groups are shown in Figure 2A. Orthogonal partial least squares-discriminant analysis (OPLS-DA) further suggested distinct metabolic profiles between the favorable and unfavorable outcome groups (Figure 3B; *R*^2^*X* = 0.156, *R*^2^*Y* = 0.712, *Q*^2^ = 0.085) (Figure 2B).

As shown in Figure 2D, five significantly differential metabolites were identified (VIP > 1, *p* < 0.05). Among them, 11-eicosenoic acid, 3-hydroxyisovaleric acid, and docosahexaenoic acid were significantly upregulated, whereas 2-hydroxycinnamic acid and cis-caffeic acid were markedly downregulated in the unfavorable outcome group. All five metabolites belonged to the organic acid class. Hierarchical clustering based on Pearson correlation coefficients further confirmed distinct serum metabolite profiles between the two groups, consistent with the PCA and OPLS-DA results (Figure 2C).

### 3.3 Comparison of serum lipidomics of ischemic stroke patients

Because serum TG levels differed between the two groups, we performed lipidomic analysis to characterize serum lipid composition. A total of 739 lipid species were detected, including 269 triacylglycerols (TAGs), 107 phosphatidylcholines (PCs), 91 acylcarnitines (AcCas), 50 phosphatidylethanolamines (PEs), and other lipid classes (Figure 3A). Relative quantification by lipid class showed substantial alterations in serum phospholipids in the unfavorable outcome group. Levels of lysophosphatidylcholine (LPC), lysophosphatidylethanolamine (LPE), PC, alkyl-ether-linked phosphatidylcholine (PC(O)), and alkyl-ether-linked phosphatidylethanolamine (PE(O)) were significantly reduced compared with the favorable outcome group (Figure 3D). In contrast, serum AcCas and fatty acids (FAs) were markedly elevated in the unfavorable outcome group (Figure 3C). Among glycerolipids, TAG levels showed an increasing trend in the unfavorable outcome group (*p* = 0.0627) (Figure 3E), consistent with the biochemical findings. No significant differences in sphingolipid levels were observed between groups (Figure 3F).

**Figure 4:**
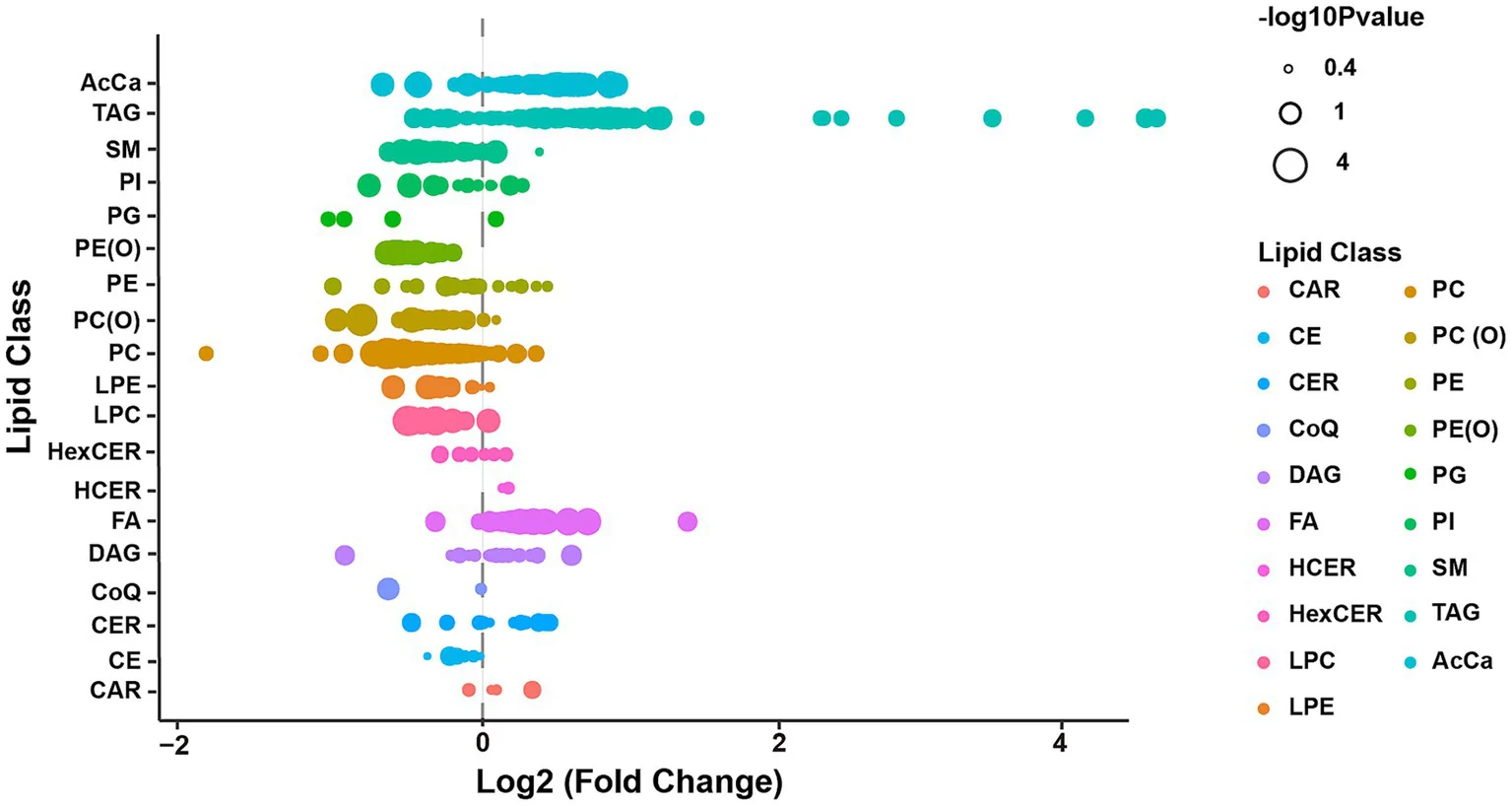
Differential serum lipid species between patients with unfavorable outcome and favorable outcome of ischemic stroke. Log2 fold changes in lipid species in the unfavorable outcome versus favorable outcome groups and the corresponding significance values displayed as –log10 (*p* value). Each dot represents a lipid species, and the dot size indicates significance.

We next examined differences at the individual lipid-species level. PCA showed clear separation of serum lipid metabolic profiles between the two groups (Figure 3B). Comparative lipidomic analysis identified 112 differential lipid molecules in the unfavorable outcome group compared with the favorable outcome group (*p* < 0.05; Figure 4). Acylcarnitines are central mediators of fatty acid transport and also contribute to metabolic regulation, disease diagnosis, and cellular signaling (18). Compared with patients with favorable prognosis, those with unfavorable outcome showed significant alterations in 19 serum acylcarnitine species (*p* < 0.05). Most medium-chain (C8–C12), long-chain (C13–C20), hydroxylated, and dicarboxylated acylcarnitines were increased in the unfavorable outcome group, whereas only C13:2, C22:3, and C8:2-OH(3) were decreased (Supplementary Figure S1).

**Figure 5:**
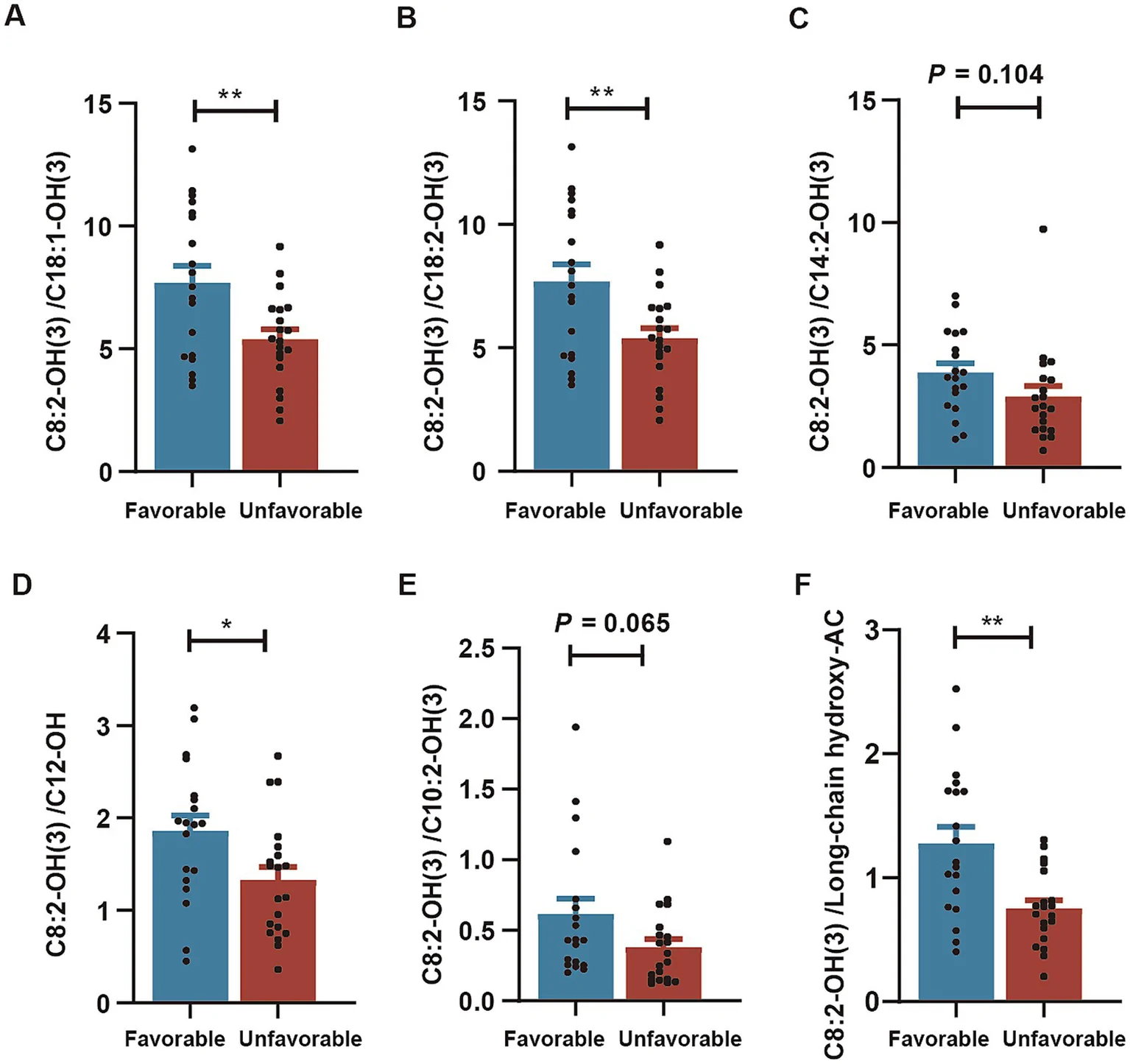
Comparison of medium-chain to long-chain hydroxy-acylcarnitine ratios between patients with favorable and unfavorable outcome. **(A)** C8:2-OH(3)/C18:1-OH(3), **(B)** C8:2-OH(3)/C18:2-OH(3), **(C)** C8:2-OH(3)/C14:2-OH(3), **(D)** C8:2-OH(3)/C12-OH, **(E)** C8:2-OH(3)/C10:2-OH(3), and **(F)** C8:2-OH(3)/total long-chain hydroxy-acylcarnitines. Acylcarnitine nomenclature: “C” denotes the total carbon number of the acyl chain, the number after the colon indicates the number of double bonds, and “-OH(3)” denotes a hydroxyl group at the C3 position of the acyl chain.

Because ratios of key acylcarnitines may provide greater biological information than individual species, we further evaluated candidate indicators of chain-shortening efficiency during fatty acid *β*-oxidation. Specifically, we examined ratios of the medium-chain hydroxy-acylcarnitine C8:2-OH(3) to several long-chain hydroxy-acylcarnitines. The ratios of C8:2-OH(3)/C18:1-OH(3) (Figure 5A), C8:2-OH(3)/C18:2-OH(3) (Figure 5B), C8:2-OH(3)/C12-OH (Figure 5D), and C8:2-OH(3)/total long-chain hydroxy-acylcarnitines were significantly decreased in patients with unfavorable outcome (Figure 5F). The ratios of C8:2-OH(3)/C14:2-OH(3) and C8:2-OH(3)/C10:2-OH(3) also showed decreasing trends, although these differences did not reach statistical significance (*p* = 0.104 and *p* = 0.065, respectively; Figures 5C,E). These observations indicate a relative accumulation of medium- to long-chain and long-chain hydroxy intermediates, accompanied by depletion of medium-chain hydroxy species.

Glycerophospholipids are the predominant phospholipid constituents of cellular membranes and are essential for maintaining neuronal membrane stability, permeability, and fluidity (19). Lipidomic analysis identified 65 significantly altered glycerophospholipid species in the serum of patients with unfavorable outcome, of which 64 showed markedly reduced relative abundance. The 10 most prominently altered glycerophospholipids included four PCs, two PC(O)s, two PE(O)s, one LPE, and one phosphatidylinositol (PI) (Supplementary Figure S1B).

Among the differential PC species, several highly unsaturated lipids, including PC(38:6), PC(42:6), and PC(42:8), were decreased in patients with unfavorable outcomes (Supplementary Figure S1B). These PC species typically contain docosahexaenoic acid (DHA), eicosapentaenoic acid (EPA), arachidonic acid (AA), or other polyunsaturated fatty acyl chains. Their reduction may therefore reflect oxidative consumption of polyunsaturated membrane phospholipids and enhanced lipid peroxidation. Serum levels of multiple ether phospholipid species, including ether-linked LPC(O), PC(O), and PE(O), were also lower in the unfavorable outcome group. Ether phospholipids are highly abundant in the brain and are predominantly localized to neuronal membranes, synaptic membranes, and related structures. Their marked reduction suggests impaired membrane-associated antioxidant defense and depletion of the membrane phospholipid pool in patients with unfavorable outcome (Figure 4A).

Excess free fatty acids, particularly long-chain and polyunsaturated species, can induce cellular injury (20). Serum levels of saturated, monounsaturated, polyunsaturated, and odd-chain free fatty acids were all significantly higher in the unfavorable outcome group than in the favorable outcome group (Supplementary Figure S1D). In addition, serum levels of multiple sphingomyelin (SM) species and coenzyme Q9 (CoQ9) were decreased in patients with unfavorable outcome (Supplementary Figure S1C). SMs are essential components of membrane lipid rafts and participate in signal transduction, apoptosis, and maintenance of myelin stability (21). Most altered SM species contained long-chain or very-long-chain fatty acyl moieties, suggesting that these fatty acids may be released from SMs and contribute to the expanded free fatty acid pool. CoQ9, a lipophilic quinone and non-protein electron carrier in the mitochondrial respiratory chain, was also reduced, suggesting impaired mitochondrial electron transport chain function (22).

**Figure 6:**
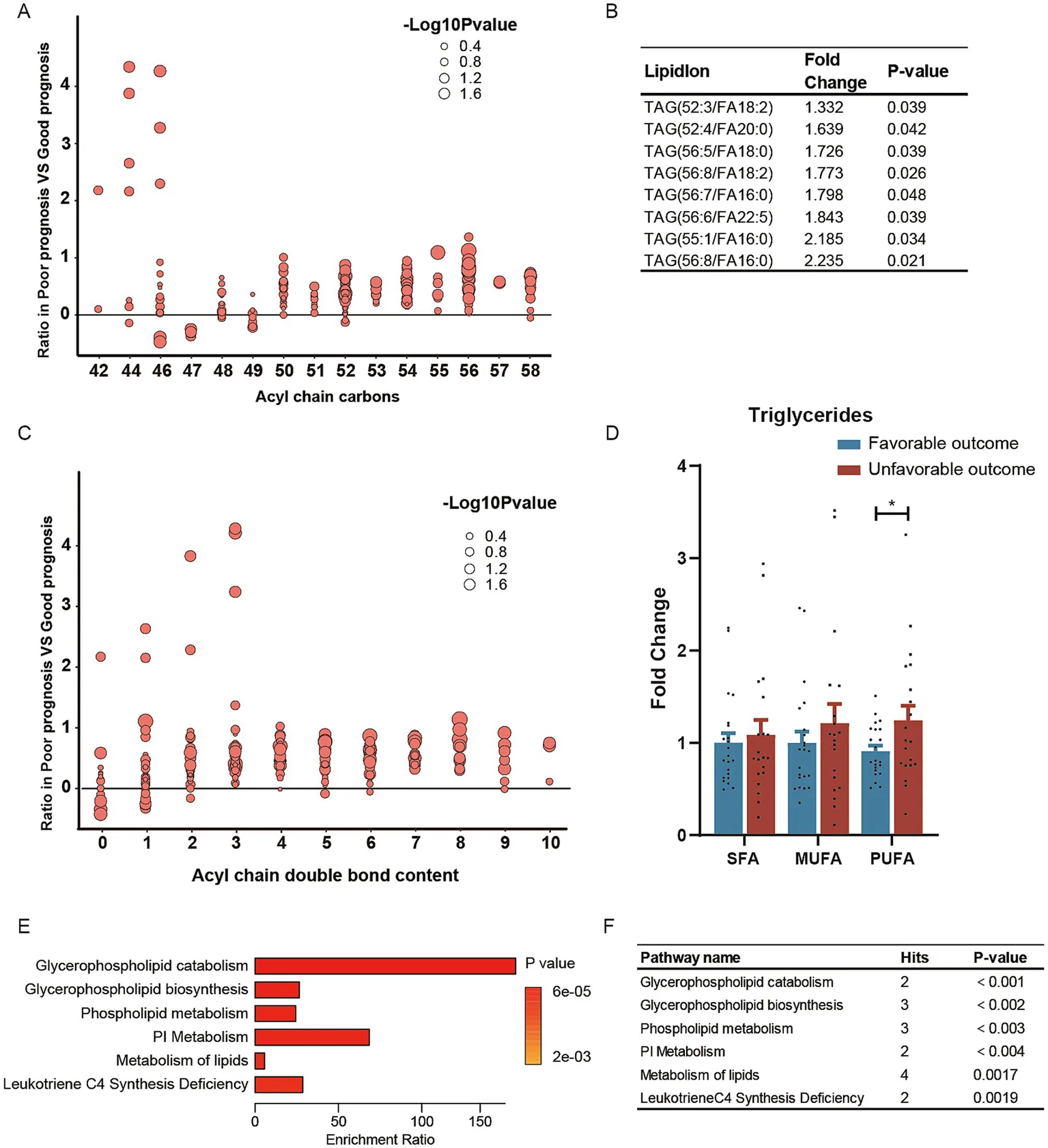
Characterization of differentially abundant triacylglycerols (TAGs) and functional pathway analysis between the favorable outcome and unfavorable outcome groups. **(A,C)** TAG pattern in the unfavorable outcome group compared with the favorable outcome group. Each dot or triangle represents a distinct TAG species, arranged along the *x*-axis according to the total number of acyl-chain carbon atoms **(A)** or the total number of double bonds **(C)**. The size of each dot or triangle is proportional to the significance value, expressed as –log10 (*p* value). **(B)** All TAG species showing statistically significant differences between the two groups (*p* < 0.05). **(D)** Serum levels of saturated fatty acids (SFA), monounsaturated fatty acids (MUFA), and polyunsaturated fatty acids (PUFA) within the TAG fraction. **(E)** KEGG pathway enrichment analysis of all differentially abundant lipid species. **(F)** Metabolic pathways exhibiting significant trends between groups.

We further compared glycerolipids, including diacylglycerols (DAGs) and TAGs. Overall, alterations in these lipid classes were limited. Among the detected TAG species, only eight showed statistically significant differences (Figure 6B). These differential TAGs were predominantly long-chain, highly unsaturated species, and their levels were increased in the unfavorable outcome group (Figures 6A,C). Consistently, these TAGs contained relatively high proportions of polyunsaturated fatty acids, whereas no significant changes were observed in saturated or monounsaturated fatty acid-containing TAGs (Figure 6D).

KEGG pathway enrichment analysis showed that the differential lipids between the poor- and favorable outcome groups were mainly enriched in glycerophospholipid biosynthesis and catabolism, the phosphatidylinositol metabolic pathway, and the leukotriene C4 synthesis deficiency pathway (Figures 6E,F). These observations suggest that lipid metabolic reprogramming may contribute to determining ischemic stroke prognosis, and the interrelated serum lipid panel identified here may represent a candidate biomarker combination for distinguishing patients with divergent outcomes.

### 3.4 Correlations of differential metabolites and lipids with clinical indicators

**Figure 7:**
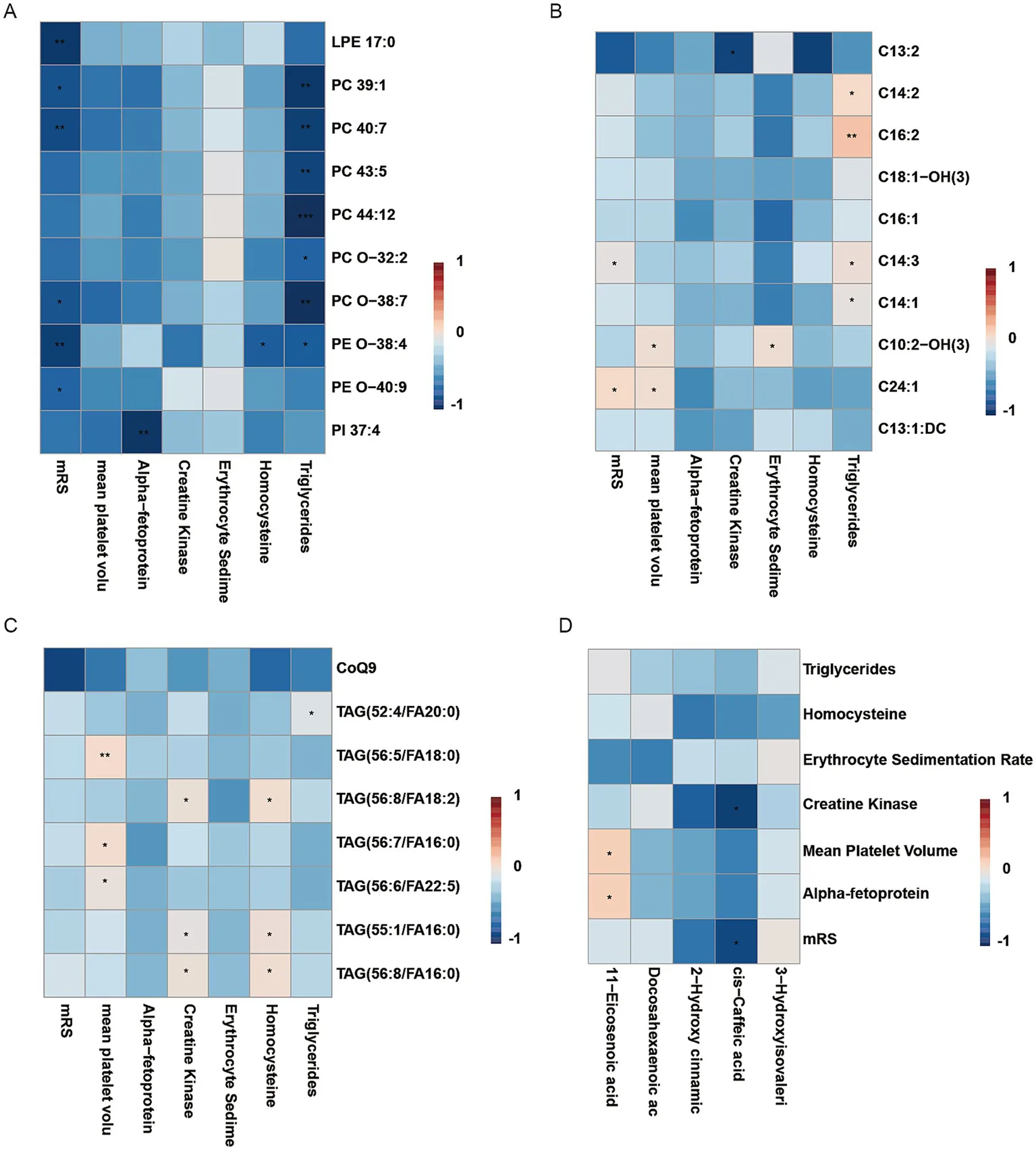
Correlation analysis between clinical biochemical parameters and differential lipids and metabolites. Heatmap showing Spearman correlation coefficients between clinical biochemical indicators and **(A)** differentially abundant phosphatidylcholines (PCs), **(B)** differentially abundant acylcarnitines (AcCas), **(C)** differentially abundant triacylglycerols (TAGs), and **(D)** differentially abundant metabolites in patients with ischemic stroke. Red denotes positive correlations and blue denotes negative correlations. The intensity of the color and the size of the squares are proportional to the correlation coefficient.

To identify molecular signatures associated with unfavorable outcome after ischemic stroke, we examined correlations between differential metabolites, differential lipids, and clinical phenotypes. Serum TG levels and mRS scores were negatively correlated with several differential glycerophospholipids and positively correlated with multiple acylcarnitine species (Figures 7A,B). Serum CK and HCY levels were positively correlated with TAG(56:8/FA18:2), TAG(55:1/FA16:0), and TAG(56:8/FA16:0) (Figure 7C). MPV was also positively correlated with several TAG species (Figure 7C). Among the differential metabolites, 11-eicosenoic acid was positively correlated with MPV and AFP (Figure 7D), whereas cis-caffeic acid was negatively correlated with CK levels and mRS scores (Figure 7D).

**Figure 8:**
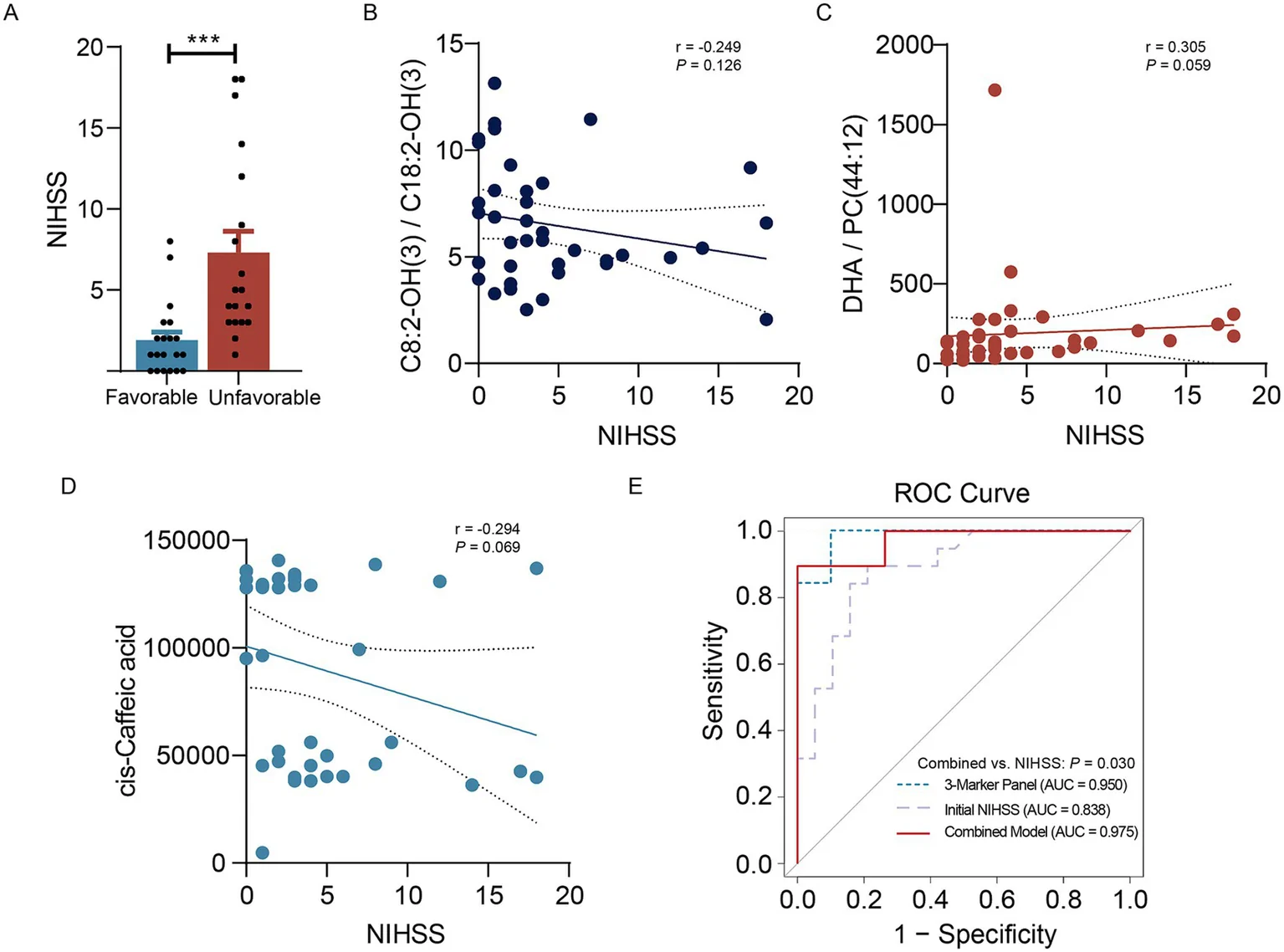
Incremental prognostic value of the three-marker metabolic panel beyond initial stroke severity. **(A)** Comparison of baseline National Institutes of Health Stroke Scale (NIHSS) scores between patients with favorable and unfavorable functional outcomes. The initial NIHSS score was significantly higher in the unfavorable outcome group. **(B–D)** Spearman correlation analysis between the initial NIHSS score and the three core metabolic markers: The C8:2-OH(3)/C18:2-OH(3) ratio **(B)**, the free DHA/PC(44:12) ratio **(C)**, and cis-caffeic acid **(D)**. The solid lines represent the linear regression fit, and the dotted lines indicate the 95% confidence intervals. The lack of significant correlations (*p* > 0.05 for all) indicates that these systemic metabolic alterations are independent of the initial acute infarct severity. **(E)** Receiver operating characteristic (ROC) curves comparing the predictive performance for unfavorable outcomes. The combined model (red solid line, AUC = 0.975) integrating the initial NIHSS score and the three-marker panel demonstrated improved discriminative ability compared to the initial NIHSS score alone (purple dashed line, AUC = 0.838) (DeLong’s test, *p* = 0.030).

Furthermore, we assessed the correlation between the three core metabolic markers and initial stroke severity to determine whether they provide independent biological information. Spearman correlation analysis revealed no statistically significant correlations between the initial NIHSS score and the C8:2-OH(3)/C18:2-OH(3) ratio (*r* = −0.249, *p* = 0.126), the free DHA/PC(44:12) ratio (*r* = 0.305, *p* = 0.059), or cis-caffeic acid (*r* = −0.294, *p* = 0.069) (Figures 8B–D). The lack of significant correlations indicates that these metabolic alterations are not merely redundant secondary reflections of the acute clinical deficit, as measured by the initial NIHSS score. These observations suggest the involvement of distinct systemic pathological processes that may influence the long-term recovery trajectory and provide prognostic information beyond the initial neurological deficit.

### 3.5 Biomarkers for the prediction of unfavorable outcome in ischemic stroke

To evaluate the predictive value of differential metabolites and lipids for distinguishing patient outcomes, we first constructed several models using 10-fold cross-validation. Receiver operating characteristic (ROC) curves showing the predictive performance of the selected biomarkers are presented in Supplementary Figures S2A–E. The model incorporating all six differential biochemical parameters achieved an area under the ROC curve (AUC) of 0.842, with a sensitivity of 0.750 and an accuracy of 0.750. The model including all five differential serum metabolites yielded an AUC of 0.750, with a sensitivity of 0.682 and an accuracy of 0.674. For models based on the 10 most differential lipid species within each lipid class, the glycerophospholipid-based model achieved an AUC of 0.725 (sensitivity = 0.705, accuracy = 0.718), the acylcarnitine-based model achieved an AUC of 0.825 (sensitivity = 0.718, accuracy = 0.719), and the TAG-based model achieved an AUC of 0.635 (sensitivity = 0.551, accuracy = 0.559).

Because these preliminary models included relatively large biomarker panels and are impractical for clinical translation, we next sought to optimize the model by focusing on key molecules mechanistically linked to the top-enriched dysregulated pathways identified in the initial discovery phase. Specifically, the accumulation of long-chain hydroxy-acylcarnitines and depletion of medium-chain species pointed to *β*-oxidation blockade, captured by the C8:2-OH(3)/C18:2-OH(3) ratio. The global decline of polyunsaturated phospholipids alongside elevated free DHA suggested structural lipid disassembly, represented by the free DHA/PC(44:12) ratio. In parallel, the depletion of cis-caffeic acid, which negatively correlated with CK and mRS scores, indicated impaired systemic antioxidant defense. Together, these three indicators represent core pathological processes associated with unfavorable outcome after ischemic stroke and have strong translational potential. We therefore integrated them into a joint prediction model. This three-marker model demonstrated promising discriminatory performance in this derivation cohort, with an AUC of 0.950, a sensitivity of 0.897, and an accuracy of 0.902.

To evaluate the added clinical benefit of our metabolic panel over conventional clinical assessment, we compared its predictive performance with the baseline NIHSS score (Figure 8A). The initial NIHSS score alone demonstrated typical predictive ability for unfavorable outcomes, yielding an AUC of 0.838 (accuracy = 0.775, sensitivity = 0.769). In comparison, our three-marker panel achieved a notably higher absolute AUC of 0.950 (accuracy = 0.902, sensitivity = 0.897), which showed a strong trend toward statistical superiority over the NIHSS score alone (DeLong’s test, *Z* = 1.911, *p* = 0.0561). When the initial NIHSS score was combined with the three-marker panel, the overall predictive capacity improved to an AUC of 0.975. DeLong’s test indicated that this combined model provided a statistically significant improvement in predictive accuracy compared to using the initial NIHSS score alone (*Z* = 2.167, *p* = 0.0302) (Figure 8E). However, given the limited sample size of our cohort (*n* = 44), the exceptionally high AUC of the combined model carries an inherent risk of overfitting and should be interpreted as an exploratory proof-of-concept. The precise incremental prognostic value of this panel necessitates stringent confirmation in larger, independent prospective cohorts.

In a multivariable logistic regression model adjusting for age and initial NIHSS score, the three-marker composite score remained significantly associated with unfavorable functional outcome (*p* = 0.0245), supporting its independent prognostic utility.

## 4 Discussion

Ischemic stroke is a leading cause of death and disability worldwide, and many survivors experience severe sequelae that markedly impair quality of life (23). Although neurological scales and neuroimaging are widely used, accurate prognostic prediction remains challenging, particularly because up to 50% of patients may lack specific early imaging abnormalities (11). Ischemic stroke may also reflect a local manifestation of systemic metabolic dysregulation involving long-term disturbances in glucose, lipid, and amino acid networks (24). Conventional lipid markers, such as TGs, total cholesterol (TC), and low-density lipoprotein cholesterol (LDL-C), do not fully capture stroke risk or prognosis. These limitations highlight the need to integrate clinical parameters with more refined metabolomic and lipidomic biomarkers.

Among routine biochemical parameters, serum HCY was significantly elevated in the unfavorable outcome group. HCY is an independent risk factor for vascular endothelial injury and neurotoxicity and has been consistently associated with post-stroke cognitive decline (25). Hyperhomocysteinemia reflects increased oxidative stress and promotes platelet activation and coagulation factor activity, thereby facilitating thrombus formation and microvascular occlusion (26). In line with this, MPV, a marker of platelet activation, was significantly higher in the unfavorable outcome group (27). In addition, 44.44% of patients in this group had elevated serum TG levels, indicating prevalent lipid dysregulation. This finding was supported by lipidomic evidence of increased specific TAG species and globally elevated free fatty acids. The absence of abnormal AFP levels reduced the likelihood that these changes were primarily attributable to overt hepatic pathology, suggesting a stronger contribution from metabolic syndrome or stroke-induced systemic metabolic reprogramming.

To clarify the metabolic basis of these disturbances, we compared serum metabolomic profiles between outcome groups. Five metabolites were significantly altered in the unfavorable outcome group, consistent with the observed clinical phenotypes. Elevated 3-hydroxyisovaleric acid suggested impaired branched-chain amino acid catabolism and mitochondrial energy dysfunction, in line with tissue injury reflected by increased CK (28, 29). Increased 11-eicosenoic acid and docosahexaenoic acid (DHA) indicated abnormal accumulation of free fatty acids, providing molecular evidence of increased lipotoxic burden (30, 31). Because DHA is highly unsaturated, an expanded pool of free DHA may provide additional substrates for lipid peroxidation and thereby exacerbate systemic oxidative stress (32). By contrast, cis-caffeic acid and 2-hydroxycinnamic acid, two phenolic acids with antioxidant activity, were significantly decreased, suggesting a reduction in systemic antioxidant reserves. This combined pattern of enhanced oxidative substrate availability and weakened antioxidant capacity provides a molecular explanation for the elevated oxidative stress suggested by increased HCY levels.

Membrane phospholipids enriched in polyunsaturated fatty acids are major targets of oxidative stress (33). Lipidomic analysis revealed a global decline in multiple phospholipid classes, including PC, PE, PI, and SM, in patients with unfavorable outcomes. Highly unsaturated PC species enriched in DHA and arachidonic acid, such as PC(38:6), PC(42:6), and PC(42:8), showed the most pronounced decreases. These phospholipids are highly enriched in neuronal synaptic membranes and myelin sheaths, and their depletion in serum is hypothesized to reflect accelerated neural membrane catabolism (34). Concordantly, free DHA was elevated in the unfavorable outcome group. The reciprocal decrease in esterified DHA and increase in free DHA provides a systemic profile consistent with the hypothesis of accelerated membrane lipid degradation. To quantify this process, we introduced the free DHA/PC(44:12) ratio. PC(44:12) is a DHA-rich, brain-enriched phospholipid, and its decline indicates consumption of DHA-containing membrane phospholipids. This ratio was significantly elevated in the unfavorable outcome group and may serve as an integrative indicator of the structural disassembly–lipotoxic burden axis.

After stroke, neurons, glial cells, and vascular endothelial cells undergo extensive membrane damage, and efficient repair depends on the availability of PC, PE, LPC, and LPE as structural substrates (35). The widespread decrease in PC suggests insufficient membrane lipid repair and remodeling, which may limit neural network recovery. Concurrent global reductions in LPC and LPE further support substrate depletion within the membrane remodeling pathway. Reduced LPC levels are often observed under severe inflammation and metabolic stress, suggesting that patients with unfavorable outcomes may experience more intense post-stroke systemic inflammation, potentially contributing to LPC pool depletion and impaired brain repair (36).

Multiple ether phospholipid species, including LPC(O), PC(O), PE(O), and plasmenyl-phosphatidylethanolamine (PE(P), plasmalogens), were also significantly reduced. Ether phospholipids, particularly plasmalogens, are abundant in neuronal membranes, synaptic membranes, and myelin sheaths. They act as endogenous antioxidants, maintain membrane fluidity, support synaptic function, and regulate inflammation (37). Their reduction may reflect an impairment of intrinsic membrane antioxidant defense (38). Together with the decreases in cis-caffeic acid and 2-hydroxycinnamic acid, these findings suggest a reduction in systemic and membrane-associated antioxidant reserves, accompanied by persistently increased lipid oxidative stress.

Widespread membrane disruption releases large amounts of free fatty acids, which can be directed toward mitochondrial *β*-oxidation or re-esterified into TAG. Serum acylcarnitine profiling showed that most medium- and long-chain, hydroxylated, and dicarboxylated acylcarnitines were significantly elevated in patients with unfavorable outcomes, whereas C8:2-OH(3) was selectively decreased. To quantify this β-oxidation blockade, we introduced the C8:2-OH(3)/C18:2-OH(3) ratio. This ratio was significantly decreased and presents a pattern that is mechanistically compatible with impaired flux through the medium-chain acyl-CoA dehydrogenase step. This profile suggests impaired mitochondrial clearance of excess fatty acids, which may exacerbate the accumulation of lipotoxic intermediates. Long-chain acylcarnitines, such as C16:1, C18:1, and C20:1, not only indicate abnormal energy metabolism but may also contribute to neurovascular unit injury (39). Collectively, this acylcarnitine profile suggests profound mitochondrial energy failure.

Corroborating this interpretation, 11 free fatty acid species, including saturated, monounsaturated, polyunsaturated, and odd-chain fatty acids, were globally elevated in the unfavorable outcome group, a profile consistent with substrate accumulation in the setting of impaired β-oxidation. Notably, several highly unsaturated TAG species, such as TAG(56:8), were increased, suggesting that when mitochondrial oxidation is impaired, excess fatty acids are diverted to the endoplasmic reticulum and sequestered through compensatory esterification to mitigate lipotoxicity (40).

These molecular alterations were closely associated with clinical phenotypes. Serum TG levels and mRS scores were negatively correlated with glycerophospholipids and positively correlated with acylcarnitines, providing clinical support for a coordinated process involving membrane phospholipid consumption and lipotoxic substrate accumulation. Cis-caffeic acid was negatively correlated with both CK and mRS, linking antioxidant defense depletion to tissue injury severity and neurological outcome. The positive correlation between 11-eicosenoic acid and MPV suggests that free fatty acid accumulation may promote platelet activation. In addition, positive correlations of CK and HCY with highly unsaturated TAG species support the presence of enhanced compensatory esterification under impaired oxidative conditions. These associations connect molecular-level disturbances with routinely available clinical parameters and offer a biological rationale for the integrated prognostic model.

Based on the three interrelated pathological features identified—structural lipid disassembly, mitochondrial energy failure, and antioxidant depletion—we constructed a parsimonious joint model using three mechanistically distinct indicators: the C8:2-OH(3)/C18:2-OH(3) ratio (reflecting *β*-oxidation blockade), the free DHA/PC(44:12) ratio (quantifying lipid disassembly), and cis-caffeic acid (indicating antioxidant depletion). This three-marker model showed promising discriminatory performance in this derivation cohort, with an AUC of 0.950, sensitivity of 89.7%, and accuracy of 90.2%. This performance substantially exceeded that of single-class models (AUC: 0.635–0.842) and conventional clinical parameters alone (AUC: 0.842). The value of this model lies not only in its predictive accuracy but also in the biological interpretability of its components, each of which corresponds to a potentially modifiable pathological process.

Several alternative explanations for the observed serum metabolic and lipidomic alterations should be considered. First, dietary habits and fasting status at the time of blood collection may influence circulating levels of exogenous metabolites such as cis-caffeic acid, as well as free fatty acids. Second, systemic inflammation, which is prevalent in the acute phase of ischemic stroke, can independently modulate hepatic lipid synthesis and secretion, potentially contributing to the altered phospholipid and triglyceride profiles observed (41). Third, pre-existing metabolic conditions—including metabolic syndrome, insulin resistance, non-alcoholic fatty liver disease, and pre-existing dyslipidemia—can produce lipidomic signatures that overlap with those attributed here to stroke-induced metabolic reprogramming (42). Fourth, renal and hepatic function can affect the clearance and interconversion of circulating acylcarnitines and fatty acids (18). Fifth, medications such as statins, antihypertensives, and antiplatelet agents may directly or indirectly influence the measured lipid species (18). Sixth, although all patients in our cohort were uniformly ineligible for acute reperfusion therapy, unmeasured differences in baseline stroke severity, including exact infarct volume, and subsequent acute medical management may also confound the metabolic readouts. Finally, the variable time from symptom onset to blood sampling introduces temporal heterogeneity, as metabolite and lipid concentrations evolve dynamically during the acute and subacute phases of stroke. While several of these factors—including admission glucose levels and pre-admission medication use—were balanced between outcome groups in our cohort, residual confounding cannot be excluded. Furthermore, the observed metabolic alterations may represent consequences of more severe initial brain injury rather than independent drivers of poor recovery. Future studies should incorporate detailed dietary and lifestyle assessments, comprehensive metabolic phenotyping, and standardized sampling time points to disentangle stroke-specific metabolic alterations from background systemic metabolic variation.

Of note, although a high baseline NIHSS score is a strong predictor of unfavorable outcome (AUC = 0.838), our three-marker panel achieved substantially higher discriminative performance (AUC = 0.950). Furthermore, integrating the initial NIHSS score with these metabolic biomarkers maximized the global discriminatory ability of the model, achieving a highly encouraging AUC of 0.975. The absence of significant correlations between these core biomarkers and the admission NIHSS score (all *p* > 0.05) suggests that these molecular alterations are not merely redundant secondary reflections of the acute clinical deficit (as measured by initial NIHSS). Rather, lipotoxicity, mitochondrial energy failure, and oxidative stress provide independent biological information regarding the patient’s long-term recovery trajectory. This highlights the substantial incremental prognostic value of our panel, enabling the early identification of vulnerable patients whose systemic metabolic collapse might not be fully captured by initial clinical scales alone.

By integrating serum metabolomics and lipidomics, this study identified global metabolic reprogramming in patients with unfavorable outcomes after ischemic stroke, characterized by structural lipid disassembly, mitochondrial energy dysfunction, and antioxidant depletion. A joint model based on three indicators reflecting these alterations—the C8:2-OH(3)/C18:2-OH(3) ratio, free DHA/PC(44:12) ratio, and cis-caffeic acid—showed promising discriminatory performance in this derivation cohort (AUC = 0.950). These preliminary findings provide a proof-of-concept for integrating molecular phenotyping with clinical assessment for early prognostic stratification and identify potential targets for interventions directed at lipotoxicity, mitochondrial dysfunction, and oxidative stress. External validation in prospective multicenter cohorts is essential.

## 5 Limitations of this study

This study has several limitations. First, owing to the exploratory nature of this high-dimensional omics study, nominal *p*-values were used without stringent false-discovery rate correction; the identified metabolic alterations are therefore preliminary and hypothesis-generating. Second, the modest single-center sample (*n* = 44) and post-hoc marker selection introduce an inherent risk of feature-selection overfitting, and the absence of an independent external validation cohort limits clinical applicability. Third, we lacked systematic data on infarct volume and stroke etiology (e.g., TOAST subtypes), which may confound the observed metabolic associations. Fourth, our lipidomic platform provides only sum-composition data (total carbon and double-bond numbers); therefore, the exact fatty acyl composition of lipids such as PC(44:12) and the stereochemistry of acylcarnitine isomers remain unconfirmed. Fifth, exogenous metabolites such as cis-caffeic acid may be influenced by diet, gut microbiota, and fasting status, while systemic liver/kidney function and inflammation may affect lipid profiles independently of neural injury. Finally, the observational design precludes causal inference, and the proposed mechanisms require validation in experimental models.

## Data Availability

The data presented in this article are not publicly available due to privacy or ethical restrictions. Requests to access the data should be directed to the corresponding author.
