# Supplementary material for: Identification of a novel metabolic and lipidomic signature for 90-day functional outcome after ischemic stroke: an exploratory proof-of-concept study
Source: Front Neurol. 2026 Sep 3;17:1931495. doi: 10.3389/fneur.2026.1931495 (PMC13581690; doi:10.3389/fneur.2026.1931495)
Supplement: Supplementary file 1 [file Data_sheet_1.DOCX]

S1

**Fig. S1 Flow diagram of patient screening and cohort derivation.** (A) A total of 146 patients with cerebrovascular disease were screened between January 2020 and December 2023. After applying predefined exclusion criteria (detailed in the diagram), a final cohort of 44 patients with acute ischemic stroke was included for untargeted metabolomic and lipidomic profiling.

S2

**Fig. S2 Top 10 most significantly altered lipid species within each lipid class between the favorable outcome and unfavorable outcome groups.** (A) Acylcarnitines. (B) Phosphatidylcholines (PC). (C) Sphingomyelins (SM). (D) Free fatty acids (FA). Lipid species are ranked by statistical significance

S3

**Figure S3. Receiver operating characteristic (ROC) curves of various models for discriminating patients with unfavorable versus favorable outcome after ischemic stroke.** (A) Clinical biochemical parameters. (B) Differential serum metabolites. (C) Top 10 differentially abundant glycerophospholipids. (D) Top 10 differentially abundant acylcarnitines. (E) Differentially abundant triacylglycerols (TAGs). (F) Joint model combining the C8:2-OH(3)/C18:2-OH(3) ratio, the free DHA/PC(44:12) ratio, and cis-caffeic acid.

Table S1 Instrument conditions for the analysis of lipidomics

| Instrument conditions | Sciex UPLC coupled to the Q-Trap 5500 mass spectrometry system | |  |  | |  |  |  |
| --- | --- | --- | --- | --- | --- | --- | --- | --- |
| Spectrum Column | BEH C8 (2.1×100 mm, 1.7μm) |  | |  | |  |  |  |
| Mobile Phase | A: Mobile phase A (%)  Acetonitrile/water (6:4, v/v)  Containing 10mM ammonium acetate | |  |  | |  |  |  |
|  | B: Isopropanol/acetonitrile (9:1, v/v)  Containing 10mM ammonium acetate | |  |  | |  |  |  |
| Gradient Profile |  |  | |  | |  |  |  |
|  | Time  (min) | Percentage  A (%) | | Percentage  B (%) | | Flow rate  (mL/min) |  |  |
|  | 0 | 68 | | 32 | | 0.26 |  |  |
|  | 1.5 | 68 | | 32 | | 0.26 |  |  |
|  | 15.5 | 1 | | 85 | | 0.26 |  |  |
|  | 18 | 3 | | 97 | | 0.26 |  |  |
|  | 18.1 | 68 | | 32 | | 0.26 |  |  |
|  | 21 | 68 | | 32 | | 0.26 |  |  |
|  |  |  | |  | |  |  |  |
| Injection Volume | 2 μL |  | |  | |  |  |  |
| Equipment parameters | Ion Spray Voltage = 500V |  | |  | |  |  |  |
|  | Source temperature = 550 °C | |  |  | |  |  |  |
|  | Turbo gas = 60 psi |  | |  | |  |  |  |
|  | Nebulizer gas pressure = 60psi | |  |  | |  |  |  |
|  | Curtain gas = 35 psi |  | |  | |  |  |  |
|  | Collision-induced dissociation (CID): Medium | |  |  | |  |  |  |
|  | Capillary temperature (℃) = 320 |  | |  | |  |  |  |
|  | Polarity: Positive/Negative |  | | |  |  |  |  |
|  | Collision Energy = 40V |  | | |  |  |  |  |
